# How to start with hip arthroscopy in a safe and effective manner, using an evidence-based approach

**DOI:** 10.1051/sicotj/2024031

**Published:** 2024-09-19

**Authors:** L. Follet, V. Khanduja, G. Thevendran, O. Ayeni, S. Shanmugasundaram, M. Abd El-Radi, H. Said, A. Abdelazeem, P. Slullitel, O. Marin-Peña, E. Audenaert

**Affiliations:** 1 Department of Orthopedic Surgery and Traumatology, Ghent University Hospital Corneel Heymanslaan 10 9000 Ghent Belgium; 2 Department of Trauma and Orthopedics, Addenbrooke’s Hospital, Cambridge University Hospitals NHS Foundation Trust Hills Road Cambridge CB2 0QQ UK; 3 Mount Elizabeth Novena Hospital 38 Irrawaddy Road Singapore 329563 Singapore; 4 Department of Orthopaedics, Institute of Clinical Sciences, Sahlgrenska Academy, Gothenburg University Gothenburg 413 45 Sweden; 5 Department of Orthopedics, Sri Lakshmi Narayana Institute of Medical Sciences Puducherry 605502 India; 6 Department of Orthopaedic Surgery and Traumatology, Faculty of Medicine, Assiut University Assiut 71515 Egypt; 7 Department of Orthopaedics and Traumatology, Kasr Alainy Hospital, Cairo Univerity Cairo 11562 Egypt; 8 The Institute of Orthopaedics “Carlos E. Ottolenghi”, Italian Hospital of Buenos Aires Buenos Aires C1181ACH Argentina; 9 Orthopaedic Surgery and Traumatology Department, University Hospital Infanta Leonor Madrid 28031 Spain; 10 Department of Human Structure and Repair, Ghent University Corneel Heymanslaan 10 9000 Ghent Belgium

**Keywords:** Hip arthroscopy

## Abstract

Hip arthroscopy is a rapidly evolving field in orthopedics, offering diagnostic and therapeutic benefits for a range of hip pathologies. This review outlines a comprehensive guide to initiating hip arthroscopy safely and effectively using evidence-based practices. Optimal surgical outcomes depend on correct indications for surgery, in particular in the presence of borderline dysplasia and degenerative joint diseases. Proper patient counseling and setting realistic expectations are crucial for satisfactory outcomes and recovery. Physical examination, radiographs, MRI, and CT scans are essential for accurate diagnosis. In case of diagnostic uncertainty, the use of intra-articular injections can help confirm the diagnosis before surgery. Techniques for hip arthroscopy include central compartment first, peripheral compartment first, and outside-in approaches. Each technique has advantages, and the optimal approach depends on the specific case. Finally, Proper operating room setup, meticulous patient positioning, and precise portal placement are critical for a successful procedure. A thorough understanding of the safe zone anatomy for portal placement is essential to minimize the risk of neurovascular complications. In conclusion, this manuscript provides a detailed, evidence-based framework for starting hip arthroscopy, emphasizing the importance of technical proficiency, patient selection, and a multidisciplinary approach to ensure patient safety and procedure efficacy.

## Introduction

Hip arthroscopy is a procedure still gaining in popularity, since 2009 the number of hip arthroscopies increased by 335–600% in the following five years [1, 2]. Because of the growing interest and developments, the management of femoro-acetabular impingement (FAI) is at a tipping point. We are shifting from a procedure performed by the pioneers and rapid adopters to a more broadly distributed usage of the technique and a more evidence-based approach. The rapid adoption of new procedures is now supported by stronger evidence, moving away from individual case reports or retrospective analyses to longer-term, prospective cohort trials [3].

Despite the growing interest in hip arthroscopy, the procedure is not one for the inexperienced surgeon. They need to master technical skills, the diagnostics, the pathophysiology, and the surgical indications. A multidisciplinary approach (physiotherapist, radiologist, orthopedic surgeon, and sports physician) is recommended for a safe arthroscopic procedure. As a surgeon, the technical skills can be optimized through a cadaver skills lab, simulation training, fellowship young adult hip, and a mentored independent practice. Hip arthroscopy is a procedure with a prolonged learning curve. Hoppe et al. saw a significant reduction in operation time and complication rates after 30 [4]. The learning curve could not be validated for 30 or any number of cases. The take-home message is: Master the technical skill, understand the pathology, and know your indication.

## Pathophysiology

**Figure 1:**
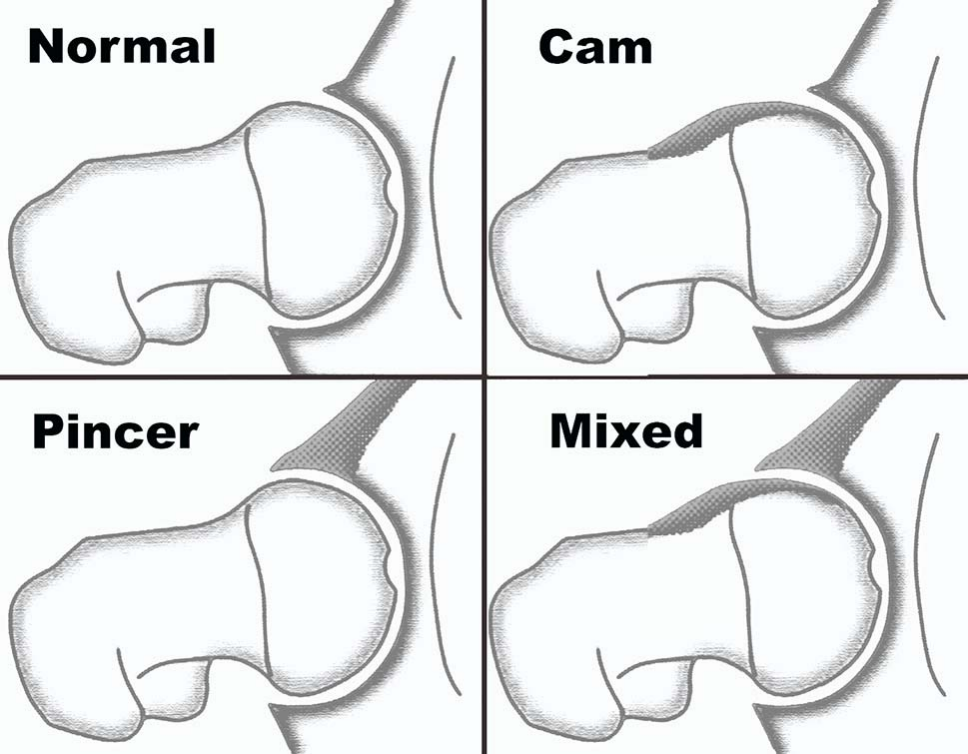
Anatomic variances leading tot femoro-acetabular impingement [6].

FAI is abnormal/premature contact of the proximal femur and the acetabulum, which can cause cartilage and labral damage and cause symptoms. These lesions can proceed in further degeneration and eventually in premature osteoarthrosis [5]. We can discriminate 3 forms of FAI: CAM impingement, pincer impingement, and combined impingement (Fig. 1) [6].

### Cam impingement

Originally described as the pistol grip deformity by Stulberg et al. [7], is a decreased anterior femoral offset caused by a bony deformation of the anterolateral side of the femoral head/neck junction. The origin of the CAM can be idiopathic (femoro-acetabular retroversion, decreased pelvic incidence, genetic) or secondary (sports participation, slipped capital femoral epiphysiodesis, leg calve perthes, trauma, osteochondroma, infection, and osteoarthrosis) [6, 8, 9].

### Pincer impingement

Over-coverage of the femoral head by the acetabulum can be caused by acetabular retroversion, which is more frequent in the female population. This is an over coverage of the femoral head anteriorly and an undercoverage posteriorly. Other causes are coxa profunda, protrusion acetabuli, and an overcorrecting peri-acetabular osteotomy (PAO). Because of the lever function, there is an impaction of the posteroinferior cartilage [6, 8, 9].

### Mixed type

In 90% of the cases, there is a combined CAM-pincer combination.

### Spinopelvic influence

The movement of the lumbar spine is in close connection with the movement of the hips especially in the sagittal plane. Patients with symptomatic FAI have less flexion in the spine and more in the hips. A lower pelvic incidence (PI) causes a decreased pelvic back tilt and accentuates contact between the acetabulum and the femur [10].

## Evidence-based medicine approach to hip arthroscopy surgical indication

**Figure 2:**
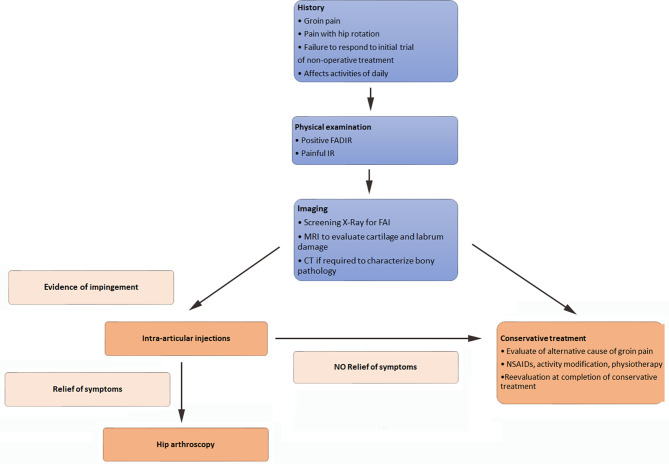
Algorithmic approach for a safe arthroscopic indication [6].

An optimal surgical result can be achieved by a good indication. Hip pain multiple DD (musculoskeletal, genitourinary, gynecological, neurological (L1L2L3) abdominal (appendicitis/hernia). Essentially everything starts with the physical examination. Upon inquiry groin dominant hip pain is important. The physical hip exam can be broken down into five parts; standing, seated, supine, lateral decubitus, and prone. Exams can include but are not limited to evaluating gait, alignment, strength, range of motion, palpation, version, and provocative testing. Upon physical examination, Flexion ADduction Internal Rotation (FADIR) is widely known. This test can be used as a screening tool when positive further examinations are needed. Radiographs are followed with MR to evaluate intra-articular damage. A CT can be valuable in evaluating bone deformations. The radial oblique sequence in the arthro mri is important in evaluating intra-articular pathology. For intra-articular problems, Arthro mri is superior to arthro CT, and arthro Ct superior to normal MRI 3T. Regarding the current investigations, a diagnostic arthroscopy is obsolete. When an intra-articular pathology is suspected an intra-articular injection is recommended. If the diagnostic infiltration gives no relief, the likelihood of relief in surgery decreases. This diagnostic algorithm is summarized in Figure 2 [6].

Satisfying postoperative outcomes is the result of the proper indication setting. This is best achieved by a team approach: the cooperation of the surgeon with the physiotherapist, radiologist, and sports physician. It is not only important to know the indications, but also the limitations of a hip arthroscopy (Table 1).

**Table 1:** Indications for hip arthroscopy.

Intra-articular indication	Extra-articular indication
Femoro-acetabular impingement (FAI)	Snapping hip
Hip dysplasia	Trochanteric bursitis
Ligament teres injuries	Gluteus medius tears
Chondral defects in the hip joint	
Septic arthritis of the hip	
Loose bodies	
Acetabular labral tears	
Synovial abnormalities of the hip	

## Hip biomechanics in femoro-acetabular impingement (FAI): how to improve with hip arthroscopy

### Patient selection

When evaluating a patient with suspect FAI we will first look for a cam lesion on imaging to decide whether there is a clinically relevant cam. So, what is a clinically relevant cam lesion? Notably, impingement occurs with different portions of the cam during different activities of daily living (ADLs), and contact studies have demonstrated all cam lesions have the potential to be harmful [5].

In addition to cam morphology, assessment of overall hip geometry is equally crucial. Both acetabular and femoral morphotypes play a critical role in this regard. Any acetabular geometry that brings the acetabulum in closer proximity to the femoral neck increases the risk of cartilage damage, making deep socket and acetabular retroversion warning signs [11]. Similarly, any configuration that brings the femoral neck near the acetabulum raises the risk of harmful abutment. As such, both excessive femoral varus and femoral version have radiographic implications [12].

In summary, the decision to operate on cam morphology necessitates a comprehensive evaluation of cam, acetabular, and femoral geometry, all of which must be assessed in a 3D context.

### Installation: the suction seal

To access the hip joint, a significant amount of traction force is necessary. To mitigate the risk of skin lesions and necrosis, a large, well-padded post will be utilized to distribute the traction forces. In addition, the Trendelenburg position can be employed to pull against gravity rather than pulling the patient against the post. The average force required for hip traction is approximately 714 Newtons, which is substantial [13].

The question of why the hip joint is so strongly sealed is seldom asked, but it may be a crucial factor in maintaining joint health. When walking, the weight of an average leg is roughly 15 kg, which translates to 150 N of force during simple ambulation. When this weight is combined with velocity, the distracting forces become even greater, with nearly 500 N of force during follow-through in soccer and up to 1000 N during a giant swing in gymnastics. Thus, the seal is a critical element in joint stability.

While a simplified vacuum model is frequently employed to explain the suction force involved in hip traction, the physics is more complicated than that. A crucial component herein involves wet adhesion. The mechanism of wet adhesion in synovial joints refers to the interaction of synovial fluid that forms a thin film with the cartilage surface [14]. This film acts as a reversible adhesive that can bond and debond with the cartilage surface depending on the applied load and shear stress. It is important for joint functioning because it prevents the joint surfaces from separating under mechanical stress. The mathematics behind this model is intricate, but several key points stand out in comparison to the simple vacuum model.

Firstly, the required force decreases as progressively more liquid bounds are broken [15]. Therefore, taking one’s time when applying traction can reduce the overall force required and the risk of skin tears. Secondly, the available joint surface is much more important than in a simplified vacuum model, with a quadratically higher impact on the force required for traction. Over-trimming should therefore be avoided, and torn labrum should be repaired as the available surface is critical to joint well-being and function. Finally, the properties of the synovial fluid, such as its viscosity and composition, can affect the force required to distract or separate the joint surfaces. Healthy synovial fluid will provide a stronger suction force, meaninga healthy jointdistracts much harder.

### Hip joint stress following cam resection

Although there is significant evidence linking femoro-acetabular impingement (FAI), particularly cam lesions, to the development of hip osteoarthritis (OA), it remains difficult to demonstrate that a well-performed cam resection can prevent the occurrence of hip OA. Biomechanics can be utilized to assess the potential impact of hip arthroscopy, and research has shown that restoring femoral sphericity is crucial for normalizing joint pressures [16, 17]. However, there is a cautionary note for dysplasia. When the femoral head or acetabulum is not intrinsically spherical, reorienting the acetabulum can decrease cartilage loading but may not fully normalize it [18].

### Importance of the hip capsule in joint kinetics

The ligaments and capsule are crucial components of the hip joint, and play a vital role in maintaining joint stability and endurance. The capsule not only restricts the joint from reaching extreme positions but also functions as a spring, assisting in returning the joint to its original position. The human body is highly energy-efficient, with approximately 70% of the energy cost for walking and running coming from elastic structures [19]. The strongest ligament in the body, the iliofemoral ligament, is a part of the anterior capsule. Research has shown that this ligament contributes significantly to walking and running, with approximately half the effort required during these activities. Therefore, when managing patients, especially athletes requiring physical endurance, it is important to minimize capsule damage and respect the role of the ligaments in maintaining physical endurance in the long term.

## Operating room setup and portals to success in hip arthroscopy

Proper patient positioning and portal placement are important for an effective and safe hip arthroscopy. This is because of the proximity of neurovascular structures. There are two main patient positioning options: the supine position and the lateral decubitus position, each with its benefits and drawbacks. In the supine position, the non-operative limb is abducted to 45°, while the operative limb is, in order, abducted, traction given, internally rotated, and then adducted on the perineal post, to achieve both longitudinal and lateral distraction, as evidenced by the vacuum sign on the C-arm image. The advantages of the supine position include the familiar orientation of the joint for the surgeon and the ability to use a routine fracture table, while the disadvantages include difficult maneuverability in obese patients and decreased posterior access [20].

In contrast, the lateral decubitus position requires a specialized traction table. The advantages of this position include easy maneuverability in obese patients and better access to the posterior and inferior joint spaces. However, this position requires extra time and a special traction table.

**Figure 3:**
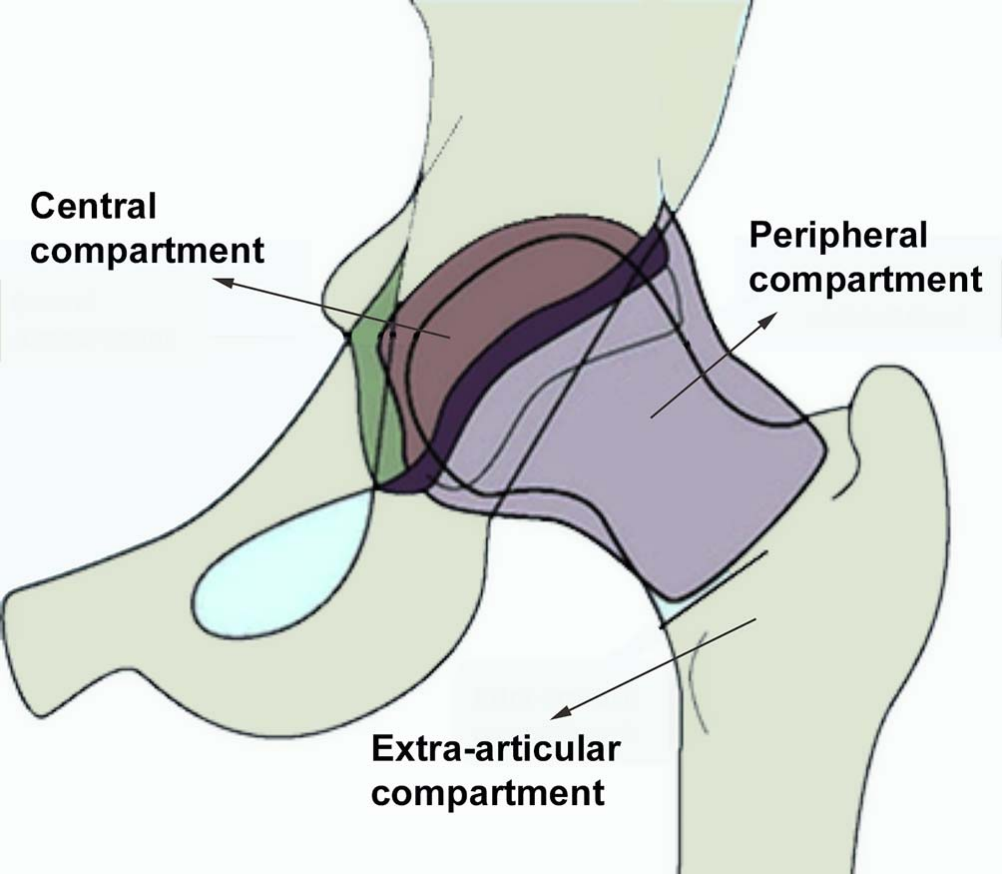
Different compartments of hip arthroscopy [21].

In and around the hip there are 3 specific regions: 1) the central compartment, 2) the peripheral compartment (which are separated from each other by the margin of the acetabular labrum), and 3) the extra-articular compartment (peri trochanteric space) (Fig. 3) [21].

Based on which compartment, the arthroscope reaches first, the method of hip arthroscopy can be classified into three techniques: 1) a central compartment first technique, 2) a peripheral compartment first technique, and 3) an outside-in technique.

The central access first technique requires fluoroscopic guidance and adequate joint distraction, and provides direct access to the central compartment. However, it has certain drawbacks like longer traction time and hence higher risk of neuropraxia and the possibility of injury to the labrum or the cartilage.

The peripheral access first technique, popularized by Dienst et al., involves arthroscopic access to the anterior femoral neck region without traction, followed by entry into the central compartment under vision and traction [22, 23]. Flexion of the hip produces relaxation of the anterior capsule and allows easy scope entry into the central compartment. The advantages of the peripheral access first technique include a lower risk of injury to the labrum and cartilage, easier and safer access, less traction time, and usefulness when central access fails.

The outside-in technique involves access to the anterior extra-capsular space under fluoroscopy, followed by an anterior capsulotomy to access the peripheral compartment. The advantages of the outside-in technique include usefulness when intra-articular access is difficult due to adhesions, while drawbacks include being a technically demanding and extensive procedure relative to the other techniques, fluid extravasation, and risk of dislocation in shallow acetabulum and in the presence of ligament laxity.

Finally, it is important to note that there are several important portals to access the hip joint. Furthermore, it is important to note the safe zone: a rectangular area drown from ASIS to the posterior side of the greater trochanter and extending 5 cm more distal.

**Figure 4:**
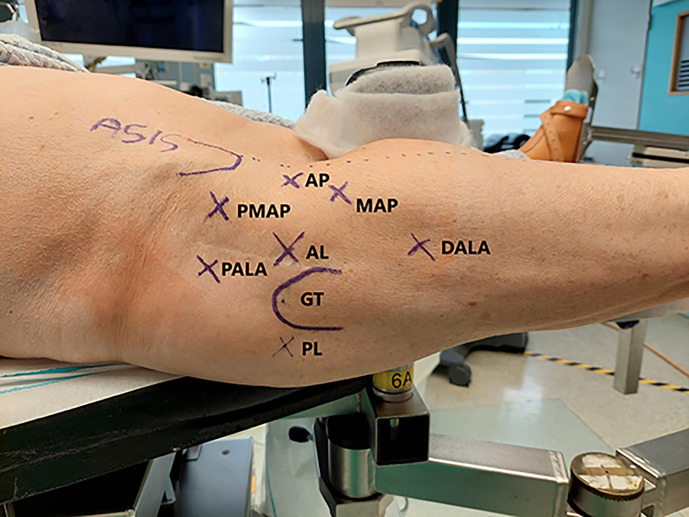
Portals of hip arthroscopy: Anterior Portal (AP), Anterolateral Portal (AL), Modified Anterior Portal (MAP), Proximal Mid Anterior Portal (PMAP), Proximal Accesory anterolateral portal (PALA), Distal Accessory Anterolateral portal (DALA) and posterolateral Portal (PL).

Some of the important portals are listed here (Fig. 4) [20, 23, 24]:

1. Anterolateral (AL) portal – 1 cm superior and 1 cm anterior to the tip of the greater trochanter
2. Anterior Portal (AP) – 1 cm lateral to the anterior superior iliac spine (ASIS) in line with the AL portal
3. Mid anterior/ Modified anterior portal (MAP)- Created by making an equilateral triangle between AL portal and anterior portal
4. DALA portal – Created by drawing an isosceles triangle between the MA and the AL portals and a point distal but in line with the AL portal
5. PALA portal – Junction of medial 1/3 and lateral 2/3 of line between the ASIS and the greater trochanter (GT)
6. Posterolateral portal (PL) – lies posterior of the top corner of the grater trochanter, passes through gluteus medius and minimus before arriving at the hip capsule.
7. Proximal Mid Anterior Portal (PMAP) – created making an equilateral triangle between Anterolateral and anterior portal to proximal (±1 cm distal of the vertical line through the ASIS).

## Arthroscopic normal view and instruments to better achieve our goal in hip arthroscopy

A safe and effective hip arthroscopy starts with the adequate tools for your surgery. Following instruments are needed when starting with hip arthroscopy

- Traction table
- Well-padded post
- Cannula for water flow
- 70° lens
- Camera
- Cannulated needle 14 gauge and nitinol wire
- Cannulated switching stick
- Half pipe
- Cannulas 4.5 and 5.5 mm
- Intra-articular knife
- Shaver

Arthroscopic evaluation of the hip joint includes two sections that can be defined as the peripheral and central compartments [25].

The peripheral compartment can be assessed without traction and in different degrees of flexion to relax surrounding ligaments and musculature [26, 27]. A variety of portals have been used. Most often, proximal and distal anterolateral portals with the 70° lens. We prefer to start with the distal anterolateral portal for peripheral compartment assessment [28, 29]. Anteriorly, the femoral neck can be evaluated and is covered by a thin periosteal layer.

**Figure 5:**
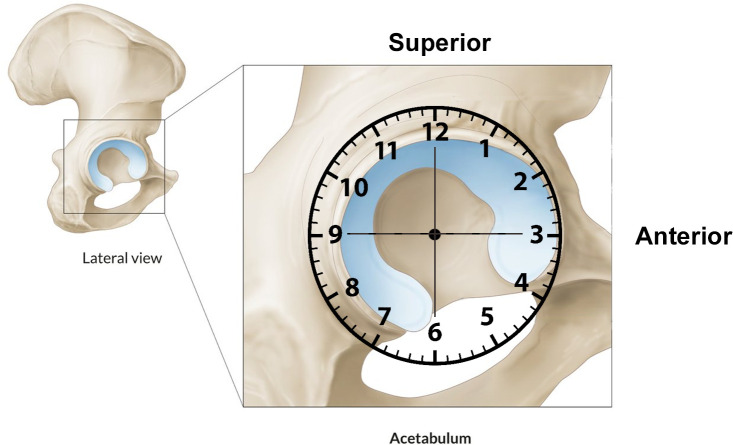
Schematic drawing of the clockwise referencing of the acetabulum [31].

**Figure 6:**
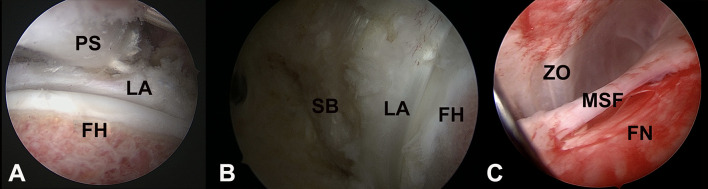
(A) Peripheral hip compartment assessment indicates femoral head (FH) and labrum (LA) as well as the Psoas tendon (PS). (B) Visualization of the subspinous bump/paralabral recess (SB), labrum (LA) and fhemoral head (FH). (C) Inspection of the femoral neck (FN), zona orbicularis (ZO) and medial synovial fold (MSF).

The 7-step routine evaluation of the hip joint is performed as described by Dienst, starting with the labrum; paralabral sulcus, medial synovial fold (MSF), Zona orbicularis, femoral neck and fat pad, femoral head, lateral synovial fold, and the psoas tendon. We propose the Said 9-step evaluation: which adds dynamic FAI evaluation and Labral probing [30]. Dynamic FAI evaluation of any Cam lesion is performed initially in 30 degrees of flexion with external/internal rotation then in further degrees of flexion as far as the traction system will allow usually 70–90 degrees. Anatomic landmarks are important to localize the pathology in the peripheral compartment. Referencing is done clockwise manner (Fig. 5). Taking the lateral hip at 12 o’clock and anterior at 3 o’clock in both hips. The subspinous bump is between 1 and 2 o’clock, the psoas tendon between 2 and 3 o’clock, and the MSF at 4 o’clock. Most of the cartilage lesions and labral lesions are located between 12 and 3 o’clock and the posterolateral impingement at 10 and 11 o’clock. Posteriorly, the reflected joint capsule with its vascular supply can be reached with flexion, adduction, and internal rotation [25]. These landmarks give excellent orientation within the peripheral compartment (Fig. 6).

Central compartment evaluation necessitates efficient traction to evaluate the space between the articular surface of the femoral head and that of the acetabulum. Central compartment is surrounded all around by the normal labrum. About 10 mm distraction is needed to allow free manipulation of 70ᵒ arthroscope and instruments [32]. For the central compartment, we add the direct anterolateral and occasionally the mid-anterior portals. With limited capsulotomies, this will provide full access to the central and peripheral compartments.

**Figure 7:**
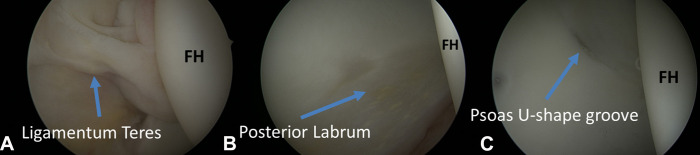
Central hip compartment assessment indicates the femoral head (FH), ligamentous teres (A), the posterior labrum (B) and the psoas U-shape groove at the 3 o’clock position.

**Figure 8:**
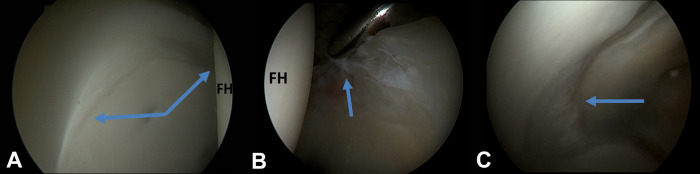
Central hip compartment assessment indicates femoral head (FH), intact chondrolabral junction with wave sign/delamination (A), labral tear with disrupted chondrolabral junction (B), acetabular fossa pointing left towards 12 o’clock (C).

As in other joints, many mapping systems have been used to divide the articular cartilage into zones. For orientation and localization, the clock face nomenclature is used [25]. In the inferior part the central hip compartment, the horseshoe-shaped lunate surface is sealed by the transverse ligament that represents 6 o’clock on clock face mapping. The acetabular fossa is filled with fat and points to 12 o’clock, which refers to lateral articular cartilage. The ligamentum teres is an around cord-like structure that extends between the cotyloid fossa and the femoral head. Anteriorly at 3 o’clock, the psoas U-shape groove is a characteristic arthroscopic landmark [25]. The labrum, and chondrolabral junction is evaluated and probed as it surrounds the bony acetabular rim and it maintains congruity of the joint (Figs. 7 and 8).

## Discussion

### When hip arthroscopy is useful in borderline dysplasia

Developmental dysplasia of the hip includes a wide spectrum of conditions ranging from a high dislocation of the femoral head to a low-volume acetabulum in the young adult. Adult hip dysplasia, therefore, is not always a straightforward diagnosis, since subtle alterations in the acetabular volume/coverage will affect the biomechanics of the hip joint resulting in hip instability and progression to intra-articular degeneration, while not producing significant initial radiological changes. Historically, a lateral center-edge angle (LCEA) below 20°, measured on an anteroposterior pelvic radiograph, has been used to categorize a dysplastic hip as such [33].

While hip dysplasia commonly corresponds with a deficient anterior-lateral wall coverage of the femoral head, one in six cases are associated with posterior wall deficiency (i.e., acetabular retroversion) [34]. It has been shown that such acetabular wall deficiencies, either anterior or posterior, may exist even with normal or so-called borderline lateral wall coverage [35]. There are also signs of missed micro-instability, i.e., upsloping of the lateral sourcil [36].

While different classification systems have been widely adopted to categorize the degree of hip dysplasia in the setting of end-stage osteoarthritis (e.g., Crowe’s or Hartofilakidis’ classifications), there are still no popularized classification systems for the pre-arthritic dysplastic hip in the young adult. Based on the LCEA, some have classified dysplasia as evident when LCEA is below 20°, and “borderline” when between 20 and 25°, considering “normal” values as those lying between 25 and 40°. While many surgeons have treated (and still treat) “borderline” dysplasia cases with hip arthroscopy alone, there is current evidence showing that such cases usually present with other features of dysplasia, and thus the terms “mild” or “borderline” may be inappropriate, with a containment procedure (i.e., periacetabular osteotomy [PAO]) better addressing the source of pain [37].

Considering that isolated analysis of the LCEA may lead to under-diagnosis of hip dysplasia, Wilkin et al., using acetabular wall indices’ measurement [38] together with LCEA and Tönnis angle measurement, have proposed a novel classification system: global/lateral dysplasia, pure anterior wall-deficient dysplasia, and pure posterior wall-deficient dysplasia [39]. The authors of the same article have reported good-to-excellent inter and intra-observer reliability using this classification [40]. With this classification, the terms “borderline” or “mild” are eliminated for their ambiguity, with hips being considered dysplastic or non-dysplastic based on 360° femoral head wall coverage analysis. Nonetheless, this classification system is based on plain radiographs only. Therefore, to better delineate the exact location of a wall deficiency, Nahal et al. have described 3D-CT acetabular wall coverage analysis with acetabular sector angle (ASA) measurement as an effective tool to characterize dysplastic acetabular morphology [41]. With this approach, anterior and posterior ASAs help the hip preservation surgeon understand the pattern of deficiency, which is essential for preoperative planning of a re-orientational procedure.

One of the case-presenters (PAS) of the past SICOT Webinar (March 30th, “360° Hip View: from Preserving to Revision THA – How to Start with Hip Arthroscopy in a Safe and Effective Manner”) presented a case of a failed borderline dysplasia initially treated with hip arthroscopy which was salvaged with a combined new hip arthroscopy + PAO. Although more evidence is still needed on the effectiveness of concomitant hip arthroscopy as an adjuvant procedure for PAO, the author of this report believes that it might be valuable in cases with previous surgery (to discard intra-articular adhesions or iatrogenic injuries), in patients, 45 years of age (to discard advanced hip arthritis) and in those with acetabular retroversion, as previously described [42]. As others have shown, PAO is, up to date, the best treatment option for so-called borderline dysplasia cases [43], and hip arthroscopy should not be used as a sole treatment for such cases, since it might increase instability and accelerate the development of secondary arthritis.

### How much degenerative changes are too much

**Figure 9:**
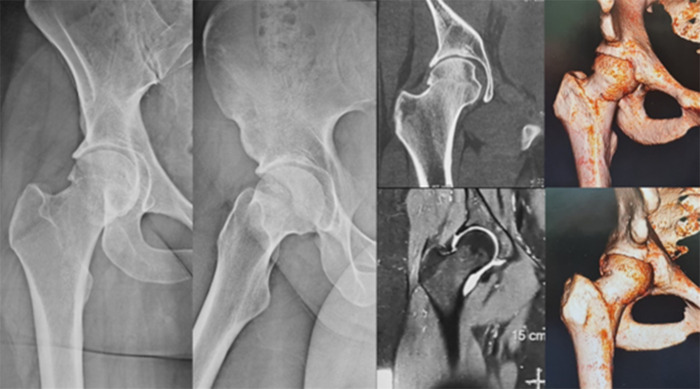
The full imaging profile needed for proper decision-making of a 28-year-old female presenting with right hip pain for two years. Plain weight-bearing radiographs (right), CT (upper left) and MRA (bottom left) reveal combined FAI with DJD.

Preservation surgery, especially arthroscopy, role in degenerative joint diseases (DJD) in all joints is a matter of high debate. Under the title “How much degenerative change is too much?”, we discussed the management of a case presentation of a 28-year-old female with normal body mass index suffering from combined femoro-acetabular impingement (FAI) with degenerative joint disease (DJD) (Fig. 9).

Preservation surgery, especially arthroscopy, in DJD in all joints depends on several factors. No doubt, its role in the late stages is negligible. However, the literature is always debatable about the early stages [44]. In the hip joint, the literature reports several factors that should be considered in decision-making and outcome expectations. Most of these factors can be gathered preoperatively from a combination of the clinical examination, especially the painful range of motion, with plain weight-bearing radiographs, computed tomography and high-resolution magnetic resonance imaging or arthrography (MRI or MRA) [45, 46].

Five factors found consensus. Patient’s presenting in the fifth decade of life and older are considered at high risk of poor prognosis, even if the arthritic changes are early. Acetabular hyaline cartilage condition is a very important factor; it depends on the degree of damage and site. In general, deep lesions with MRI Outerbridge ≥ 2 are associated with unfavourable results. Moreover, if it is located in the weight-bearing surface area, this is an additive strong poor prognostic factor contrary to the peripherally located lesion. The radiographic degree of joint arthritic changes, especially if the joint space narrowing < 2 mm or its grade is more than two, according to Tönnis, is of poor outcome. Finally, the degree of damage to the femoral head must be considered. Poor prognosis is excepted when the femoral heads show chondral lesions, subchondral cysts, and oedema [46].

**Figure 10:**
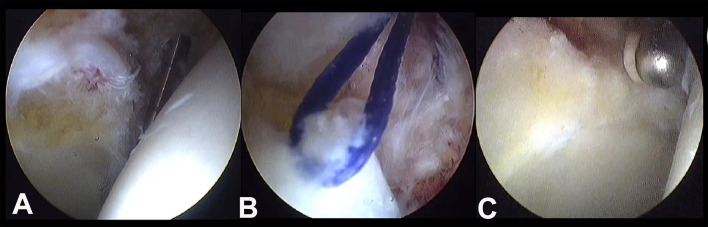
Intraoperative photos show the degenerative changes in the labrum and the peripheral hyaline cartilage (A). A trail of loop suture ripped and cut through the degenerated labrum (B). Finally, partial labrectomy and acetabulum recession were done (C).

Another matter of debate is surgical management and what to do with these hips. As preservation surgeons, we are always keen on repairing and reconstructing the damaged labrum or hyaline cartilage; however, excision and debridement are performed here more, due to their degenerative nature and fear of the persistence of pain after the operation (Fig. 10). This is combined with the classical excision of acetabular over coverage and femoral bumpectomy, if needed, to stop further mechanical damage [44–46].

Of utmost importance, the webinar panel agreed that these patients’ results are always below the level of their expectations and hopes, especially in young active ones. We recommend proper patient counselling and avoidance of false, unrealistic expectations [46, 47].

## Conclusion

Hip arthroscopy has proven to be a safe and efficient procedure for diagnosing and treating various hip pathologies, despite a significant learning curve. The procedure demands a comprehensive understanding of technical skills, diagnostics, and pathophysiology. For the starting arthroscopist, adhering to evidence-based practices and involving a multidisciplinary team enhances safety and efficacy. Proper patient selection, meticulous preoperative planning, and precise intraoperative techniques are critical. The importance of appropriate patient positioning, portal placement, and traction management cannot be overstated. By considering these aspects, hip arthroscopy can be performed safely, even by those new to the procedure.

## Data Availability

The datasets generated and analyzed during the current study are available from the corresponding author upon reasonable request. Data supporting the findings of this study may be subject to certain restrictions due to privacy or ethical considerations. Any requests for data sharing will be reviewed on a case-by-case basis in accordance with institutional guidelines.
